# Higher striatal glutamate in male youth with internet gaming disorder

**DOI:** 10.1007/s00406-023-01651-5

**Published:** 2023-07-28

**Authors:** Johanna Klar, Johannes Slotboom, Stefan Lerch, Julian Koenig, Roland Wiest, Michael Kaess, Jochen Kindler

**Affiliations:** 1 University Hospital of Child and Adolescent Psychiatry and Psychotherapy, University of Bern, Bern, Switzerland; 2 Support Center for Advanced Neuroimaging (SCAN), Neuroradiology, University Hospital of Bern, Inselspital, Bern, Switzerland; 3 Department of Child and Adolescent Psychiatry, Center for Psychosocial Medicine, University of Heidelberg, Heidelberg, Germany; 4 Department of Child and Adolescent Psychiatry, Psychosomatics and Psychotherapy, Medical Faculty, University of Cologne, Cologne, Germany; 5 Clinic and Polyclinic for Child and Adolescent Psychiatry, Psychosomatics and Psychotherapy, University Hospital Cologne, Cologne, Germany; 6 Institute of Diagnostic and Interventional Neuroradiology, University Hospital of Bern, Inselspital, Bern, Switzerland; 7 Department of Neurology, University of Zurich, Zurich, Switzerland

**Keywords:** Internet gaming disorder, Reward system, Striatum, MR-spectroscopy, Glutamate

## Abstract

Internet gaming disorder (IGD) was included in the Diagnostic and Statistical Manual of Mental Disorders (DSM-5) as a research diagnosis, but little is known about its pathophysiology. Alterations in frontostriatal circuits appear to play a critical role in the development of addiction. Glutamate is considered an essential excitatory neurotransmitter in addictive disorders. This study’s aim was to investigate striatal glutamate in youth with IGD compared to healthy controls (HC). Using a cross-sectional design, 25 adolescent male subjects fulfilling DSM-5 criteria for IGD and 26 HC, matched in age, education, handedness and smoking, were included in the analysis. A structural MPRAGE T1 sequence followed by a single-voxel magnetic resonance spectroscopy MEGA-PRESS sequence (TR = 1500 ms, TE = 68 ms, 208 averages) with a voxel size of 20 mm^3^ were recorded on 3 T Siemens Magnetom Prisma scanner. The voxel was placed in the left striatum. Group comparison of the relative glutamate and glutamine (Glx) was calculated using regression analysis. IGD subjects met an average of 6.5 of 9 DSM-5 IGD criteria and reported an average of 29 h of weekly gaming. Regression analysis showed a significant group effect for Glx, with higher Glx levels in IGD as compared to HC (coef. = .086, *t* (50) = 2.17, *p* = .035). Our study is the first to show higher levels of Glx in the striatum in youth with IGD. The elevation of Glx in the striatum may indicate hyperactivation of the reward system in IGD. Thus, results confirm that neurochemical alterations can be identified in early stages of behavioral addictions.

**Supplementary Information:**

The online version contains supplementary material available at 10.1007/s00406-023-01651-5.

## Introduction

Since internet gaming disorder (IGD) was included in the research appendix of the Diagnostic and Statistical Manual of Mental Disorders (DSM-5), a growing number of studies investigated IGD (American Psychological Association, APA, 2013). However, the pathophysiology of IGD still remains elusive. The state-of-the-art definition of IGD by the APA covers nine criteria: preoccupation, withdrawal, tolerance, unsuccessful control, loss of interest, continuation despite problems, deception, escape negative feelings, and risk opportunities. To fulfill diagnostic criteria for IGD, 5 out of 9 criteria must occur within the last 12 months.

Human behavior is motivated by reward. In addictive disorders a hyperactive reward system may override the capacity of its control system, leading to unrestrained seeking of a substance or—in the case of IGD—the uncontrolled use of online computer games [46]. The reward system includes a cortico-basal ganglia circuit, which involves the orbitofrontal cortex, anterior cingulate cortex, the ventral striatum and the ventral pallidum [15]. The ventral striatum consists of the nucleus accumbens and the ventral parts of the nucleus caudatus and the putamen [15]. Furthermore, parts of the prefrontal cortex (PFC, control, motivation), the ventral tegmental area (VTA, arousal), the amygdala (salience), and the hippocampus (memory) play important roles in maintaining neuronal balance between reward seeking and control functions in addiction [18, 46]. The ventral striatum (nucleus accumbens) is the key region of this subcircuit with dopaminergic and glutamatergic projections from all the other regions [18]. The function of the ventral striatum is to mediate goal-directed behavior, integrating information from cortical and the other limbic structures [34]. Therefore, it is central in the development of addiction.

The neurochemistry of the striatum in addiction has been targeted before, mainly using rodent models [38]. Based on the latter studies, the glutamate hypothesis of addiction has been formulated [18]: early stages of addiction are usually accompanied with high/intense consumption of a substance and concomitantly with a high release of dopamine and glutamate in the striatum [18]. Especially glutamate in the nucleus accumbens has been related to increased craving for a substance [34]. In the course of the disease, initial extensive glutamate activity may result in neuroadaptations and consequently decreased levels of glutamate in the striatum without substance intake at later stages [18, 38]. Glutamate is coupled with glutamine in a metabolic cycle [37]. Neuronal released glutamate gets converted to glutamine in astrocytes and reconverted into glutamate by a specific enzyme [37].

Magnetic resonance spectroscopy (MRS) provides information on biochemical substrates in defined brain areas, providing concentration information in the respective area. Multiple studies have been performed correlating these concentration variations to specific study conditions [44]. Previous MRS studies on glutamate in the striatum in substance addiction showed inconsistent results with no difference or reduced glutamate levels in patients with chronic forms of substance addiction in comparison to healthy controls [13, 22, 25]. These inhomogeneous results may be caused by methodological issues, such as decreasing signal to noise ratio (SNR) in subcortical as compared to cortical brain areas. Recently pulse sequences using metabolite cycling (MC) have been proposed for measuring the metabolic profile of the nucleus accumbens. More specific, studies using MC-MRS found decreased glutamate levels in chronic cocaine addiction but no differences in smoking [10, 41]. Due to the fact that the nucleus accumbens is very small, 3 Tesla (3 T) may result in too low SNR spectra in clinical acceptable time. This problem can be solved by going to ultrahigh field (7Tesla) using SLOW-EPSI sequence [49] which enables whole brain gamma-aminobutyric acid (GABA+) and glutamate editing having a spatial resolution in which the nucleus accumbens can resolved within 10 min of measurement time. With currently available 3 T MRS sequences it is technically not feasible to reliably differ between glutamate and glutamine. Therefore, glutamate is often referred as Glx, the sum of glutamate and glutamine.

However, research on the pathophysiology of addictive disorders is still largely based on substance-related addiction [47]. Yet, there may be important differences between substance and behavioral addictions, e.g. the intake of a neuroactive substance directly interacts with the release of neurotransmitters in the brain, whereas behavioral addictions may only have an indirect and subtle influence on neurotransmission. The main outcomes of previous neuroimaging research on pathological internet use indicate structural, functional and connectivity changes that lead to an imbalance of the reward and its control system [19, 48].

So far, only a few studies about neurochemical alterations in IGD have been conducted using MRS. Han et al. found in a sample of 73 patients with IGD compared to 38 healthy controls (HC) a decreased level of *N*-acetyl aspartate (NAA), measuring a voxel placed in the frontal cortex and lower levels of choline measuring a temporal placed voxel [16]. Bae et al. investigated a group of 28 youth with IGD and Attention Deficit Hyperactivity Disorder (ADHD) compared to 42 HC and found a decreased level of NAA in a frontal placed voxel [3]. In both studies the decreased NAA level in the frontal voxel was interpreted as reduced activity in frontal brain structures [3, 16]. The decreased level of choline measured in the medial temporal cortex was interpreted as indicator for a dysfunction of the medial temporal cortex in IGD [16].

To our knowledge, glutamatergic alterations in the striatum in youth with IGD were not investigated previously. Thus, the aim of this study was to measure neurochemical alterations in youth with IGD with a focus on glutamate in the ventral striatum. Based on previous research about substance-based addictions, the hypothesis was to find a deviation of glutamate levels within the striatum in adolescents with IGD compared to healthy controls.

This investigation promises insights into the neuronal mechanisms of early stages of behavioral addictions such as IGD in young male subjects and may add important new information to previously conducted MRS research on chronic substance related addictions.

## Methods

### Study description and sample

The sample included 55 male youth with an age range of 15–25 years. Twenty-nine youth with IGD (5–9 DSM-5 IGD criteria) and 26 HC (0–1 DSM-5 IGD criteria) were recruited. The groups were matched for age, educational level, handedness (right, left), and smoking status (never, occasionally, regularly). Exclusion criteria were contraindications for magnetic resonance imaging (MRI), substance abuse (excluding tobacco), medication intake affecting the hypothalamic–pituitary–adrenal (HPA) axis, chronic somatic-neurologic diseases, schizophrenia, and bipolar affective disorder, type 1. Furthermore, exclusion criteria for the HC group were any DSM-5 diagnosis according to the Mini-International Neuropsychiatric Interview (MINI(-KID), [1, 35] and higher use of online gaming than the Swiss mean (1,5 h/day during the week, 2 h/day weekends; youth, activities, media-survey Switzerland; [42]. Participants were recruited via public advertisements (e.g., online platforms, schools, and universities) in the canton of Bern. IGD subjects were additionally recruited in the outpatient units of the University Hospital of Child and Adolescent Psychiatry and Psychotherapy, Bern.

In a first step, a telephone interview was conducted with all potential participants in which inclusion and exclusion criteria were checked after providing information about the project and discussing questions about study participation. After inclusion, all participants provided written informed consent and, in case of minors, consent was additionally provided by parents. The diagnostic assessment was conducted at a first appointment. One week afterwards, the MRI measurement took place at a second appointment. During the week between the two sessions dynamic data were collected with Ecological Momentary Assessment (EMA) in the participant’s daily routine.

### Diagnostic assessment

The first appointment included the following clinical interviews and a questionnaire to assess the severity of IGD and comorbidities. According to the state-of-the-art definition of IGD by the APA, the assessment included the Structured clinical interview for IGD diagnosis (DSM-5; APA, 2013). To meet diagnostic criteria for IGD, participants needed to fulfill 5 out of 9 criteria within the last 12 months. For HC, fulfilling more than one criterion was defined as reason for exclusion. The Video Game Dependency Scale (Computerspielabhängigkeitsskala (CSAS); [33] is a self-report 18-item questionnaire that specifically captures the pathological use of internet games and was used as an additional instrument in adjunction with the clinical interview. The 18-items cover the nine DSM-5 IGD criteria and are answered on a four-point scale. The cut-off for IGD is a sum score over 16 points. The psychometric properties of the CSAS show a good construct validity (*α* = 0.95) and reliability (*r* = 0.84) [33]. The MINI(-KID) [1, 35] covers all common Axis-I psychiatric disorders, listed in the DSM-IV and International Classification of Diseases (ICD-10) and was used as a screening instrument for comorbidities. For pediatric disorders, the adjusted MINI-KID was used for the participants in the range of 15–17 years. The Beck Depression Inventory (BDI-II); [6] is a well-established self-report questionnaire and was used to assess depressive symptoms. As anxiety disorders often occur as a comorbidity of IGD, the Beck Anxiety Inventory (BAI) [5], was used to assess participant’s self-reported anxiety levels.

### Ecological momentary assessment

To collect dynamic data during the daily routine of participants, EMA was used. The EMA approach was introduced and started after the diagnostic assessment at the first appointment. A study smartphone with the movisensXS app (Movisens GmbH, Karlsruhe, Germany) was handed to every participant. Sampling points were in the morning and evening during weekdays and every hour during the day (8 a.m.–11 p.m.) on weekends (total sampling points/subject/week: max. 46). The participant was reminded in time with a push message on the study smartphone. The questions covered gaming behavior (e.g., gaming time) among other topics (e.g., emotions, stress, Internet use). Answers were provided on visual analog scales or by forced-choice answers.

### MRI data acquisition

**Fig. 1:**
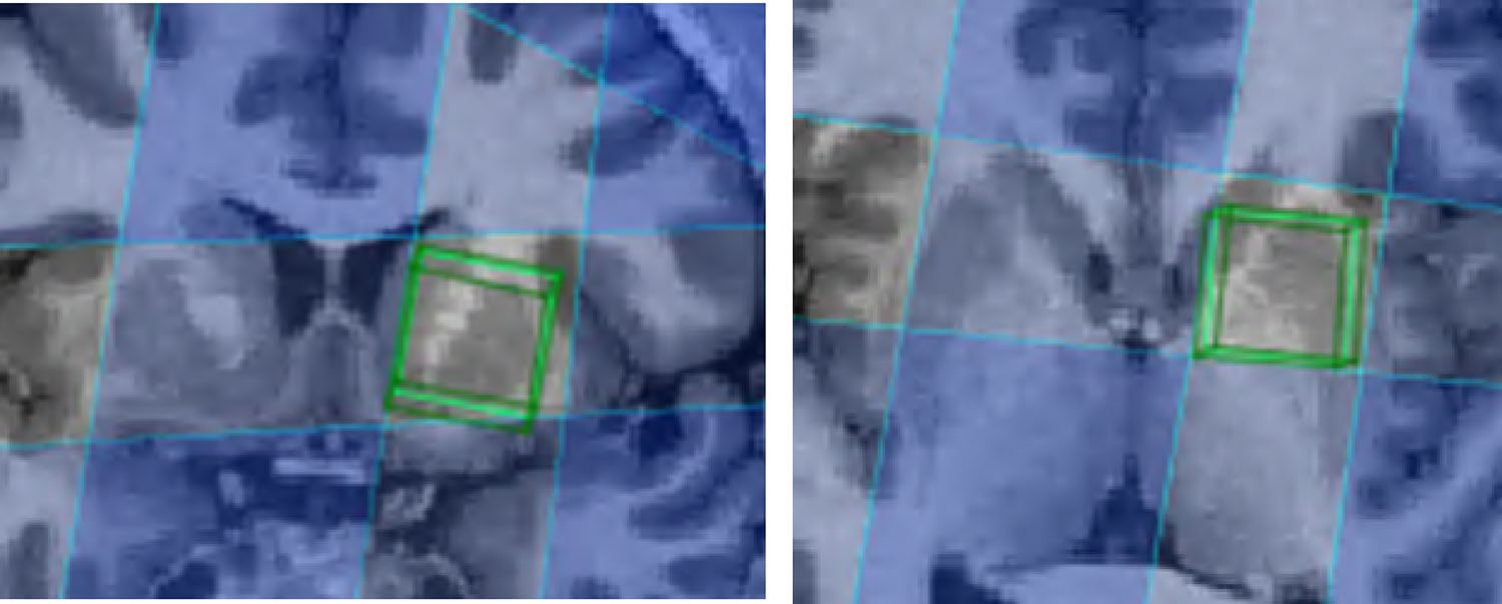
Voxel placement. T1 structural image of one representative participant using spectrIm-QMRS. The magnetic resonance spectroscopy voxel (20 × 20 × 20 mm, green box) was placed in the left striatum

After 1 week of EMA, all subjects were measured using 3 T Siemens Magnetom Prisma scanner with a 64 channel head coil located at the University Hospital of Bern (Inselspital). A structural MPRAGE T1 sequence (TR = 2300 ms, TE = 2.98 ms, TI 900 ms) was initially recorded. On the structural T1 images, the voxel was placed manually in the left ventral striatum, covering the nucleus accumbens, as shown in Fig. 1. To quantify glutamate and glutamine (Glx), one single-voxel MRS MEGA-PRESS GABA+ edited dataset was recorded for optimal measurement with GABA+ [23], including water reference signals (TR = 1500 ms, TE = 68 ms, 208 averages, bandwidth 1.2 kHz), and voxel size (20 × 20× 20 mm). The protocol that was used to perform spectral editing is svs-edit-859G. The bandwidth of the MEGA pulses was 50 Hz applied on 1.9 ppm. The svs-edit-859G WIP package provides automatic frequency control during recording to compensate for frequency drift during the recording of the sequence. The number of transients was 416: 208 times ON and 208 times OFF, making a complete measurement time of 10 min and 30 s.

### MRS data analysis

*Software package* MRS data preprocessing and quantification was performed using the spectrIm-quantitative-MRS (QMRS) plugin [30] of the JAVA-based Magnetic Resonance User Interface (jMRUI) software package [39]. The preprocessing of the edited and non-edited spectra included water removal, apodization, and offset correction in the frequency domain. First, the water signal was removed using Hankel-Lanczos singular value decomposition filtering (HLSVD; [31]). The spectra were smoothed with a 3 Hz Gaussian filter, and offset deviations were corrected. In the non-edited spectra, frequency shift correction was applied additionally. For the edited and non-edited spectra, time-domain-frequency-domain-(TDFD)Fit quantification models were defined, applying maximal prior knowledge [36].

*Basis sets* Similar to LC-Model, TDFDFit enables the fitting of spectra based on basis sets. For this project separate spherical phantoms containing 10 mmol pH7 phosphate buffered of creatine, NAA, GABA+, glutamate etc. were made. Individual phantom spectra were fitted with TDFDFit modelling the metabolite spectra as aggregation of Voight-lines (so-called numeric patterns). TDFDFit can use these numeric patterns to fit the in vivo spectra with. The advantage of this approach is that the basis set is generated with the same sequences as the in vivo spectra, instead of idealized simulated basis sets. This approach was necessary, since the source code of the spectral editing sequence, and therefore the timing was not known.

Glx was fitted in the edited spectra and calculated as the sum of glutamine and glutamate, as they show a strong overlap of spectral patterns [21, 32]. See Appendix 2.0. for examples of glutamate and glutamine fitting. Since Glx is prone to strong individual variations, for the analysis, the Glx/creatine (Cr) ratio was calculated because no such variability was expected for creatine and therefore suited as internal reference [21]. As a sensitivity analysis, the difference of GABA+ between groups was analyzed. To assess the quality and comparability of the spectra, voxel segmentation (grey matter, white matter, cerebrospinal fluid (CSF)) was conducted using the spectrIm-QMRS segmentation functionality. Furthermore, all data with a fit quality number (FQN) higher than 2.0 were manually checked to exclude spectra with poor quality (see Appendix [21]). The FQN was defined conform the expert consensus [29].

### Statistical analysis

Statistics were implemented using STATA SE 16.1 [40]. A two-sided significance level of *α* = 0.05 was determined for all analyses. The matching of the groups by age, educational level, and smoking status was evaluated using *t*-tests and Chi-square. A regression analysis was conducted to investigate the influence of group on Glx levels, including grey matter percentage in the voxel as control variable. Finally, Pearson correlations between the scores of the diagnostic instruments and the Glx (Glx/Cr) score were analyzed.

### Ethics

The study procedures were carried out in accordance with the Declaration of Helsinki. The study was approved by the local ethics committee of Bern (KEK 2018-01604). All subjects were informed about the study and provided written informed consent and, in case of minors, consent was additionally provided by parents.

## Results

### Demographic characteristics

The detailed demographics by group are listed in Table 1. There were no significant group differences in age, educational level, or smoking status. One single participant of the IGD group received psychotropic medication (fluvoxamine) during the study, whereas the other patients were medication naive. The IGD group fulfilled on average 6.5 of the nine DSM-5 diagnostic criteria for IGD and a general mean gaming time of 29 h per week (questionnaire). According to CSAS norms for youth and young adults (ages 16–30), the average daily gaming time of about four hours is higher than average. The sum score of 20.3 points is also above average (percentile rank 89–96).

**Table 1:** Sample demographics

	IGD	HC	Matching
*N*	25	26	
IGD criteria (mean)	6.5	0.4	
Psychotropic medication (*n*)	1	0	
CSAS (sum score, mean)	20.3	3.6	
Gaming (weekly, mean, h)	29	3.9	
Age (min–max, mean)	15–25 (20.04)	16–25 (20.65)	*t*(49) = 0.743 *p* = 0.461
Handedness (right/left; *n*)	23/2	24/2	Chi^2^ = 0.34 *p* = 0.844
Educational level (secondary I/secondary II, tertiary; *n*)	9/15/1	5/17/4	Chi^2^ = 3.05 *p* = 0.218
Smoking status (never/occasionally/regularly; n)	16/7/2	14/8/4	Chi^2^ = 4.28 *p* = 0.639
ADHD (yes/no; *n*)	2/25	0/26	Fisher's exact test *p* = 0.235

*IGD* internet gaming disorder, *HC* healthy controls, *n* number, *CSAS* Video Game Dependency Scale, *h* hours, age in years, *ADHD* attention deficit hyperactivity disorder clinical diagnosis

### Ecological momentary assessment

The overall compliance with the EMA assessments was 64.1% (IGD = 66.2%, HC = 64.1%). A total of 1461 reports of gaming time were provided by participants. IGD participants reported a mean gaming time of 17.9 h during the week of EMA measurement. HC reported a significant lower mean gaming time of 3.8 h during the week of EMA measurement (*t*(49) = − 3.62, *p* < 0.001).

### MRS voxel composition and data quality

The voxels’ grey matter, white matter and CSF were quantified with spectrIm-QMRS by segmentation of the MPRAGE T1 data. There were no significant group differences in the mean percentage of grey matter (IGD = 82.9%, HC = 80.6%, *p* = 0.073), white matter (IGD = 17.1%, HC = 19.3%, *p* = 0.108) and CSF (IGD = 0.09%, HC = 0.13%%, *p* = 0.268) of the voxel composition. Furthermore, there was no significant group difference in the mean level of noise in the spectra (*p* = 0.831). The SNR of NAA was around 22, whereas the SNR of Glx was between 2.5 and 5.0. All data with a FQN higher than 2.0 were manually checked, and four spectra from the IGD group had to be excluded due to low quality. Data points that could represent outliers were again examined with special care to exclude influences due to insufficient data quality or deviating psychopathological characteristics (e.g., ADHD diagnosis). The residuals of GABA+ were not distributed normally (S-Wilk *p* < 0.001), therefore a log transformation was conducted (S-Wilk *p* = 0.180). No group effect on GABA + was shown in the regression analysis (coef. = − 0.052, *t*(49) = − 0.35, *p* = 0.727).

### Group difference in Glx

**Fig. 2:**
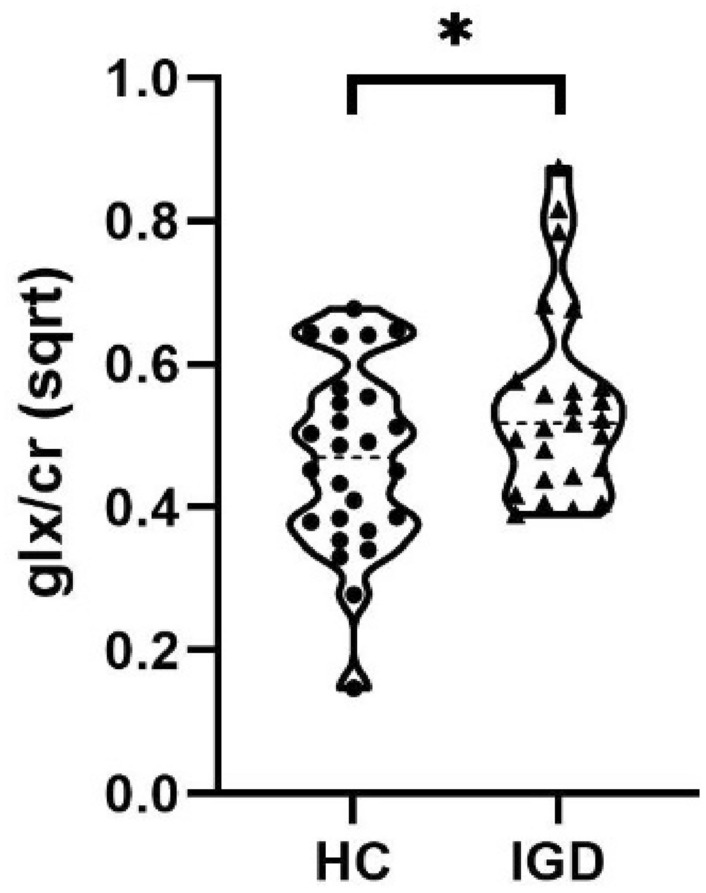
Regression analysis with the square-root transformed data showed a significant effect of group on Glx levels (Glx/Cr ratio). In the striatum a significant higher level of Glx/creatine ratio (*p* < 0.05) was detected in the IGD group as compared to HC. Mean and distribution, *HC* healthy controls, *IGD* Internet gaming disorder, *Glx* glutamate + glutamine, *sqrt* squareroot

The IGD group showed a mean Glx/Cr ratio of 0.31 (SD = 0.16) and the HC group a mean of 0.23 (SD = 0.12). The Shapiro–Wilk normality test showed a significant deviation of the Glx/Cr data from a Gaussian distribution curve (*p* < 0.001). Therefore, a square-root transformation was conducted that resulted in an approximation of the Glx/Cr data to a normal distribution (S-Wilk *p* = 0.29). Regression analysis with the square-root transformed data showed a significant effect of group on Glx levels (Glx/Cr ratio), with higher levels in the IGD group (coef. = 0.086, *t*(50) = 2.17, *p* = 0.035) compared to HC (Fig. 2). Excluding the participant with psychotropic medication did not change the results (coef. = 0.069, *t*(49) = 2.06 *p* = 0.045).

### Correlations

The results showed no significant correlations between Glx/Cr ratio and DSM-5 IGD criteria, CSAS sum score, gaming time (EMA), BDI-II score, and BAI score. Details are shown in Table 2.

**Table 2:** Correlations between glx and diagnostic instruments

Glx/Cr	IGD DSM-5	CSAS	Gaming EMA	BDI	BAI
Glx/Cr	0.23 (*p* = 0.11)	0.10 (*p* = 0.49)	0.17 (*p* = 0.23)	0.03 (*p* = 0.84)	0.001 (*p* = 0.99)

*Glx* glutamate + glutamine, *Cr* creatin, *IGD* internet gaming disorder, *CSAS* Video Game Dependency Scale (sumscore), *Gaming EMA* hours of gaming during one week measured with Ecological Momentary Assessment, *BDI* Beck Depression Inventory (sumscore), *BAI* Beck Anxiety Inventory (sumscore)

## Discussion and conclusion

To our knowledge, this is the first study showing glutamatergic alterations in the striatum in IGD. The main finding is a significantly higher level of striatal Glx in a male adolescent sample with IGD compared to a matched HC group.

These results support the Kalivas glutamate hypothesis of addiction, confirming a glutamatergic imbalance in the reward system in the early stages of behavioral addictions [18]. Glutamate seems to play an important role in the development and maintenance of addiction, especially in learning and cue-reactivity processes [20]. As described before, the striatum receives glutamatergic projections from brain areas involved in addiction: the prefrontal cortex, amygdala, and hippocampus [43]. Furthermore, there are intense dopamine projections from the ventral tegmental area to the striatum [43]. The release of glutamate in the striatum and especially the nucleus accumbens is strongly related to the dopamine transmission with reciprocal interactions [43]. Therefore, the imbalance in glutamate might be promoted by a dopaminergic imbalance, with a higher dopamine increase in the reward circuit in the early stage of behavioral addiction [50].

In the initial state of addiction, dopamine release in the PFC in a reward situation promotes glutamate release in the VTA which further promotes glutamate release in the ventral striatum [43]. Sensitization processes lead to an increased biological reaction to repetitive reward stimuli with increased direct glutamatergic input from the amygdala to the striatum [45]. The development of addictive behavior occurs through learning processes that lead to an anticipatory expectation of reward in exposure to a certain stimulus or intake of a substance. This learned reward in response to a stimulus leads to a strong cue-reactivity of the reward system. In the state of withdrawal, the reward connected stimuli become salient and the reward system shows an immediate strong reaction [43]. Glutamate gets highly released in the hippocampus, amygdala, and PFC and triggers an over-activation of the striatum, especially of the dorsal striatum which leads to a strong urge to perform the related addictive behavior [43]. In all of the described states of addiction increased glutamatergic transmission plays an important role and, thus, the study results of higher Glx levels in youth with IGD can be well integrated within previous addiction research. The finding of higher Glx levels within striatal regions also aligns with the clinical definition of IGD by the DSM-5 diagnostic criteria, e.g. the criteria preoccupation, withdrawal, tolerance, and unsuccessful control, describe excessive and uncontrollable gaming use suggest an activated reward system. The clinical definition of IGD is thereby supported by the results.

The Interaction of Person-Affect-Cognition-Execution (I-PACE) model for addictive behaviors is a popular framework for internet gaming disorder and other addictive behaviors. The I-PACE provides a theoretical basis for the development and maintenance of addictive behaviors, bringing together predisposing factors, affective and cognitive reactions to stimuli, and executive functions [9]. The I-PACE framework further specifies brain circuitries involved in the development and perpetuation of addictive behaviors, with a differentiation between the function of the ventral and dorsal striatum in this process [9]. The ventral striatum seems to be involved in an early stage of addiction as part of the hyperactive sensitized reward system, whereas the dorsal striatum seems to play a role in a later habituated stage of addiction [9, 11]. Thus, the higher Glx level in IGD found in our study could be interpreted as an indication of an overactive striatum due to addictive processes like sensitization or craving.

Previous substance-based addiction research involving human subjects, measuring glutamate in the striatum with spectroscopy, showed inconsistent results with no difference or reduced glutamate level in patients with chronic forms of substance addiction in comparison to healthy controls [10, 13, 22, 25, 41]. However, there are important differences to our study, for instance concerning the measured sample. While substance abuse disorders show the highest prevalence in adulthood, IGD is most prevalent in youth. One possible explanation for the higher level of Glx in the striatum in the studied IGD sample might be the young age and, therefore, early stage of addiction. Furthermore, the intake of a substance (e.g., cocaine) directly influences the release of neurotransmitters in the brain, whereas gaming has only an indirect influence on this release [38]. Nonetheless, both, substances and certain behaviors, lead to an activation of the reward system. Furthermore, the results showed no correlations of clinical measures such as diagnostic criteria or survey results with the Glx level in the striatum. This does not necessarily mean that there is no association between those measures, but this outcome may be due to the composition of the sample studied. As the samples were well matched but showed a clear difference in IGD symptoms, the results could be attributed to this specific distinction and might not be better explained by comorbidities or other behaviors measured in this study. Measuring a more stratified sample in a longitudinal design, including a clinical control group, might show these associations. The rather small sample size might have been a limiting factor, future studies should include bigger samples to replicate the results.

This study’s strength is a new localization of the MRS measurement, Glx in the striatum that was not measured before in IGD research. Furthermore, the two compared groups were apart from the IGD diagnosis, well-matched in age, handedness, education, and smoking status. Age, hormone status and smoking have previously been associated with brain glutamate levels (e.g. [4, 12, 17, 27]). However, the present sample consisted of young, physically healthy, and male participants, exclusively. Thus, the composition of the sample and close matching of the groups rigorously reduces confounding factors to be related to the findings of this study.

One limitation of MRS studies is the size of the measured voxel. As the voxel of the present study covered large parts of the striatum, it was not possible to discriminate between potentially different alterations in the ventral and the dorsal striatum. However, a reduction of the voxel size would have led to an unacceptable low signal-to-noise ratio (SNR). Moreover, the spectral resolution of 3 T MRS does generally not allow a reliable discrimination of glutamate and glutamine. Therefore, Glx levels (sum score glutamine + glutamate) are presented, which is in line with several recent studies investigating glutamatergic mechanisms in psychiatric disorders (e.g. [7, 8]. MEGA-PRESS MRS was originally designed to measure GABA+. However, the quantification of low concentration J-coupled metabolites such as Glx from TE 68 spectra provides reliable results and has recently been used in several studies (e.g. [24, 28, 51]). In future, MRS with higher field strength (7 T) and improved shimming techniques will allow to measure the ventral part of the striatum covering mainly the nucleus accumbens and the distinction of glutamate and glutamine [14]. Another limitation, is the missing of a sensitivity to reward measure in the study, future studies should include such a measure e.g. the sensitivity to punishment and sensitivity to reward questionnaire.

Further research should address other behavioral addictions, not only IGD, to replicate our findings. The clinical implication of the reported results is the reaffirmation of IGD as psychopathology, showing neurochemical alterations associated with the gaming behavior. Furthermore, the results could have an impact on the therapeutic approach in the treatment of IGD. Recently glutamate transmission has been considered as a possible therapeutic target for pharmacological interventions. Medications that reduce the glutamate transmission, for instance, *N*-methyl-d-aspartate receptor antagonists, could be used to reduce craving and prevent relapse [18, 38].

In summary, this is the first study showing higher levels of Glx in the striatum applying MRS in male youth with internet gaming disorder compared to a HC group, indicating an overactive reward system in early forms of IGD.

### Supplementary Information

Below is the link to the electronic supplementary material.

Supplementary file1 (PDF 1130 KB)
