# Supplementary material for: Higher striatal glutamate in male youth with internet gaming disorder
Source: Eur Arch Psychiatry Clin Neurosci. 2023 Jul 28;274(2):301–9. doi: 10.1007/s00406-023-01651-5 (PMC10914841; doi:10.1007/s00406-023-01651-5)

Appendix

1. MRS examples

1.1 Example for good quality of the spectrum, made with spectrIm-QMRS.

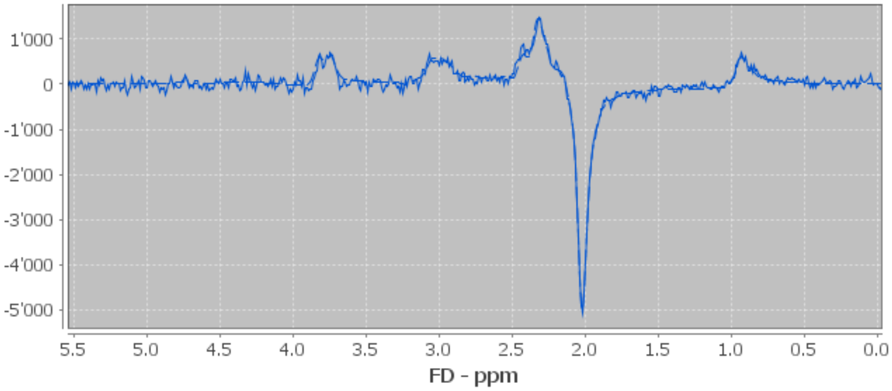

Enlarged view of a good quality spectrum, made with spectrIm-QMRS.

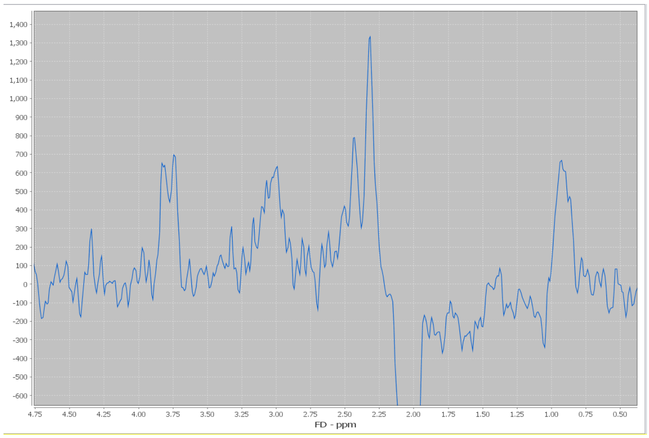

1.2 Example for bad quality of the spectrum that lead to the exclusion of the subject, made with spectrIm-QMRS.

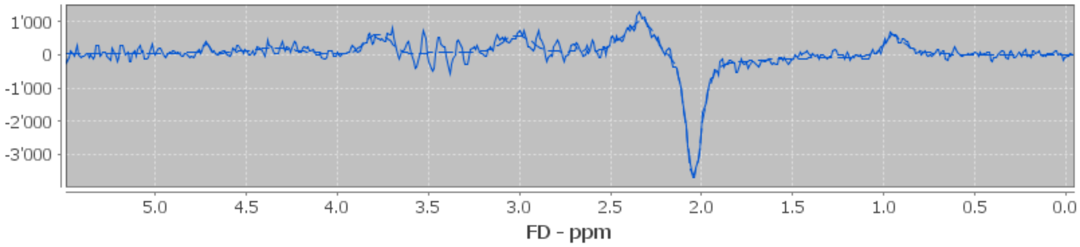

2. Glx model fitting

2.1 Model fit of glutamate (blue, made with spectrIm-QMRS.)

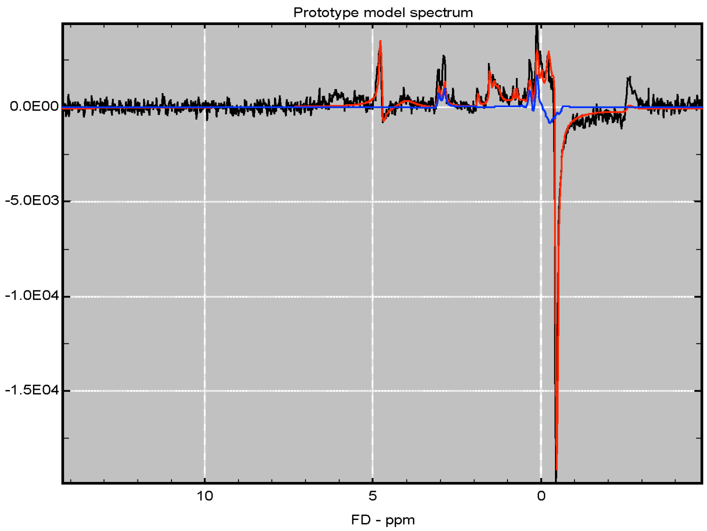

2.2 Model fit of glutamine (blue, made with spectrIm-QMRS.)

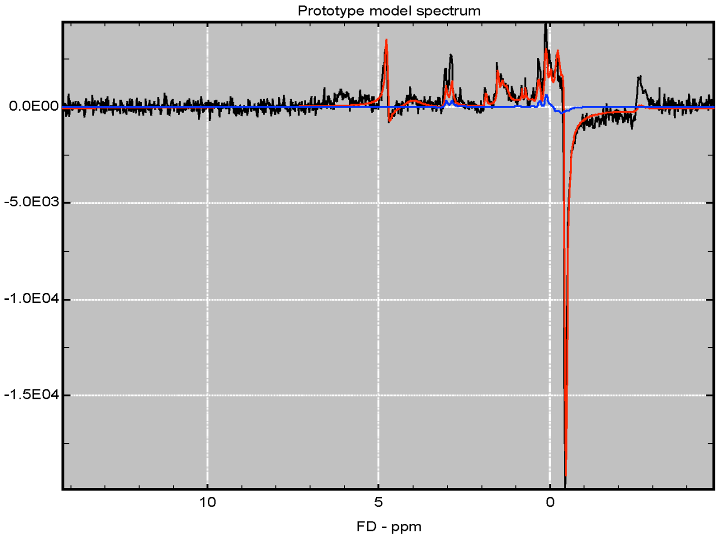

Supplement: Supplementary file 1 — Supplementary file1 (PDF 1130 KB) [file 406_2023_1651_MOESM1_ESM.pdf]
